# The interoceptive communication model (ICM 2.0): a state-dependent neurophysiological framework for behavioral and cognitive accessibility

**DOI:** 10.3389/fpsyg.2026.1884838

**Published:** 2026-10-06

**Authors:** Yoandra M. Gomez Uncu, Leydis Morejón Rodríguez

**Affiliations:** Research Department, Beautiful Minds ABA Services, Fort Myers, FL, United States

**Keywords:** interoception, physiological state, state-dependent behavior, autonomic regulation, behavioral accessibility, cognitive accessibility, emotional regulation, executive functioning

## Abstract

Behavioral, emotional, and cognitive variability across contexts remains insufficiently explained by models that rely on environmental contingencies, cognitive processes, or static trait-based constructs. Individuals may exhibit marked fluctuations in executive functioning, emotional regulation, learning access, behavioral flexibility, and adaptive responses despite retaining knowledge, insight, or previously acquired skills. These patterns are frequently reported in trauma-related conditions, chronic stress, autism spectrum disorder, psychiatric disorders, rehabilitation, and educational settings. ICM 2.0 proposes that these fluctuations reflect dynamic changes in physiological accessibility rather than permanent changes in an individual’s underlying capacities. This study operationally defines accessibility as the probability that a previously demonstrated capacity can be recruited under specified physiological, contextual, and task conditions, explicitly distinguishing it from capacity, motivation, engagement, arousal, and performance outcomes. Accessibility is conceptualized as a latent state-dependent construct inferred from converging physiological, behavioral, subjective, and, when available, neural indicators. The Interoceptive Communication Model (ICM 2.0) is proposed as a state-dependent neurophysiological framework that organizes interactions among autonomic regulation, interoceptive processing, affective signaling, sensory load, endocrine activity, immune signaling, environmental context, and higher-order regulatory modulation. The model introduces two broad processing tendencies. Route 1 reflects a limbic-autonomic survival-oriented pattern associated with restricted adaptive accessibility. Meanwhile, Route 2 reflects an insular–prefrontal regulatory pattern associated with expanded adaptive accessibility. These terms are explicitly heuristic and are not intended as anatomical or localized neural pathways. ICM 2.0 generates testable predictions: physiological dysregulation is expected to reduce access to adaptive capacities independently of prior skill acquisition, and regulation-focused interventions are expected to increase accessibility before measurable behavioral improvement from skill training alone. The framework is differentiated from Polyvagal Theory, predictive processing, active inference, and neurovisceral integration. It also specifies measurement strategies, biological mechanisms, and empirical testability criteria.

## 1 Introduction

Behavior science has historically interpreted behavior through models that emphasize environmental contingencies, cognitive mechanisms, learning history, or relatively stable trait-based constructs. These frameworks have contributed substantially to psychology, education, psychiatry, rehabilitation, and behavior analysis (Baer et al., 1968; Beck and Bredemeier, 2016; Cooper et al., 2020).

However, individuals may exhibit substantial variabilities in adaptive functioning, despite stable knowledge, motivation, or prior skill acquisition. For example, they may demonstrate flexible reasoning, emotional regulation, and social engagement in one physiological condition but exhibit rigidity, impulsivity, narrowed attention, reduced task access, and even shutdown in another. Such variability has been consistently observed across trauma-related conditions, chronic stress, autism spectrum disorder, psychiatric disorders, rehabilitation, and educational settings. Converging evidence from interoception, autonomic regulation, affective neuroscience, stress physiology, and executive-function research suggests that an individual’s physiological organization shapes their perception, attentional prioritization, emotional stability, decision-making, and behavioral readiness (Barrett and Simmons, 2015; Craig, 2009; Critchley and Garfinkel, 2017; Khalsa et al., 2018; Mather and Thayer, 2018). The existing models to interpret behavior have largely failed to explain these fluctuations within individuals under varying psychological states.

This study proposes the Interoceptive Communication Model (ICM 2.0) to addresses what it calls the “accessibility problem”: adaptive capacities may exist and be previously demonstrated but become difficult to recruit under partṣicular physiological states. The model shifts the explanatory question from whether the individual possesses the capacity to the physiological and contextual conditions under which that capacity is accessible in real time.

The revision incorporates reviewer feedback by (a) operationalizing accessibility, (b) defining the physiology–behavior relationship as a dynamic heuristic rather than a formal equation, (c) clarifying the differences between ICM 2.0 and major competing frameworks, and (d) introducing falsifiable predictions and mechanisms constraints.

## 2 Accessibility: conceptual and operational definition

The ICM 2.0 defines accessibility conceptually as the moment-to-moment functional accessibility of behavioral, cognitive, emotional, relational, and regulatory capacities under dynamic physiological conditions.

Operationally, accessibility is defined as the probability that a capacity that was previously demonstrated under low-demand or regulated conditions can be recruited under a specific physiological state, environmental context, and task demand (Gomez Uncu, 2026c). This formulation avoids circularity by decoupling accessibility from task success or failure alone. Instead, it is estimated using converging physiological, behavioral, subjective, and, when available, neural indicators obtained before or during the task.

Importantly, accessibility is not conceptualized as a separate physiological system, behavioral trait, motivational variable, or performance outcome. It is instead proposed as an emergent property of interacting physiological processes that determine the availability of moment-to-moment adaptive capacities. No single measure is considered equivalent to accessibility. It is treated as a latent state-dependent construct that must be inferred from multiple converging indicators across behavioral, physiological, cognitive, and subjective domains (Barrett and Simmons, 2015; Critchley and Garfinkel, 2017; Garfinkel et al., 2015; Khalsa et al., 2018).

This formulation distinguishes capacity from accessibility. Capacity refers to whether a skill or adaptive response exists in the individual’s repertoire. Conversely, accessibility refers to whether that capacity can be deployed in real time under current physiological and contextual conditions. For example, an individual may possess language, academic skills, or coping strategies, yet access to these capacities may vary under stress, fatigue, pain, sensory overload, threats, or dysregulated arousal (James et al., 2023; McEwen, 1998; McEwen and Wingfield, 2003).

Accessibility is distinguished from related constructs such as arousal, which reflects the level of activation; engagement, which describes observable participation; motivation, which encompasses values, preferences, or incentives; readiness, which refers to preparedness for participation; and task performance. Instead, accessibility refers specifically to the moment-to-moment, state-dependent availability of previously acquired or biologically supported capacities (Table 1).

**Table 1:** Candidate operational markers of accessibility.

Domain	Candidate markers	Accessibility interpretation	Important cautions
Autonomic regulation	Resting and task-related heart rate variability (HRV)/respiratory sinus arrhythmia, heart rate recovery, respiratory rate, skin conductance, pupil dilation, and blood pressure reactivity.	Greater flexibility and faster recovery should be associated with broader adaptive accessibility; elevated physiological load and delayed recovery should predict restricted access.	HRV is not a direct measure of accessibility; interpretation must consider respiration, medication, age, sleep, fitness, and health status.
Interoceptive processing	Heartbeat discrimination/tracking, respiratory interoception, confidence-accuracy calibration, MAIA-2, BPQ, interoceptive attention, and accuracy indices.	More coherent interoceptive monitoring and metacognitive calibration should support improved state discrimination and regulation.	Close interoceptive attention is not always adaptive; accuracy, interpretation, and regulation must be differentiated.
Stress and biological load	Cortisol awakening response, salivary cortisol reactivity/recovery, sleep duration/quality, fatigue, pain, and inflammatory markers (when feasible).	Higher cumulative biological load should constrain accessibility, particularly for executive control, learning flexibility, and emotional regulation,	These markers are temporally sensitive and require standardized sampling and context control.
Behavioral availability	Response latency, error recovery, perseveration, behavioral flexibility, variability, adaptive help-seeking, and transition tolerance.	Behavioral indicators should exhibit state-dependent changes even when underlying knowledge remains stable.	Behavioral outputs alone cannot define accessibility due to overlap with outcomes.
Cognitive and emotional tasks	Working memory, inhibitory control, set-shifting, emotion regulation tasks, frustration tolerance, and dual-task performance under physiological load.	Within-person variability across physiological states should exceed trait-based predictions of performance.	Tasks must control for fatigue, learning and task difficulty, and environmental demands.
Subjective and relational state	Self-reported perceived safety, threat, sensory load, fatigue, readiness, emotional intensity, and co-regulation availability.	Subjective states may indicate whether physiological load is experienced as a threat or be overwhelming, exhausting, or tolerable.	Self-report may be unavailable or unreliable in acute dysregulation, young children, or in some clinical contexts.
Neural indicators, when available	Insula, anterior cingulate cortex, prefrontal cortex, amygdala, brainstem activity; network-level integration; and heartbeat-evoked potentials.	Accessibility should correspond to reflect integrated brain × body regulation, rather than localized activity.	Neural measures are not required for routine clinical assessment are used as hypothesis testing tools.

This table presents candidate indicators for estimating accessibility across domains. No single indicator should be interpreted as a direct measure of accessibility. The proposed framework requires psychometric validation, reliability testing, and empirical calibration before clinical or research application as a standardized assessment (Agorastos et al., 2023; Forte et al., 2019; Garfinkel et al., 2015; James et al., 2023; Khalsa et al., 2018; Mehling et al., 2018; Mather and Thayer, 2018; Thayer and Lane, 2000).

## 3 The accessibility problem in human functioning

A central challenge across psychology, neuroscience, education, psychiatry, rehabilitation, and behavioral science is explaining why the same individual exhibits substantial variability in adaptive functioning across contexts. For example, trauma-related conditions may involve rapid shifts in defensive responding, dissociation, attentional narrowing, or emotional regulation even in objectively safe environments (Lanius et al., 2018; Lanius et al., 2020). Meanwhile, in autism and sensory-related conditions, adaptive functioning may vary with sensory load, predictability, fatigue, and autonomic reactivity (Mazefsky et al., 2013; Mazefsky et al., 2012). In executive-function difficulties, individuals may retain planning or inhibitory capacities but fail to recruit them under high physiological or cognitive load (Forte et al., 2019; Mather and Thayer, 2018). Therefore, adaptive functioning depends not only on learning history or motivation but also on the physiological organization supporting real-time access to relevant capacities.

ICM 2.0 views this through the lens of accessibility, defined as the mismatch between preserved capacities and functional access under different physiological environments. The framework conceptualizes accessibility as a dynamic, multi-system phenomenon that emerges from interactions among autonomic regulation, interoceptive signaling, affective salience, sensory integration, endocrine and immune load, environmental context, relational safety, and higher-order regulation (Barrett and Simmons, 2015; Critchley and Garfinkel, 2017; Feldman, 2017; Khalsa et al., 2018; McEwen and Wingfield, 2003; Alotiby, 2024). The framework therefore incorporates a state-dependent layer to existing models that determines the influence of physiology on the availability of adaptive capacities.

## 4 State-dependent dynamics and the nonlinear physiology–behavior equation

The original formulation of ICM 2.0 described state-dependent behavior through the Non-Linear Physiology–Behavior Equation as a conceptual representation of the dynamic relationships among antecedents, physiological state, consequences and behavioral expression (Gomez Uncu, 2026a,c):


$$\begin{array}{l} A↔P↔C\\ ↓\\ B \end{array}$$

where the antecedents (A) and consequences (C) interact bidirectionally with physiological state (P). Behavior (B) is conceptualized as the downstream expression of this dynamic organization. In this revision, the equation is explicitly defined as a conceptual dynamic-systems heuristic rather than a formal mathematical model.

Nonlinearity refers to the fact that changes in physiological state are not expected to lead to proportional behavioral effects across all contexts. Instead, the model predicts threshold effects, hysteresis, recovery lag, cumulative load interactions, and state-dependent transitions in accessibility that may occur disproportionately to changes in environmental demand. A threshold effect occurs when a small increase in physiological load produces disproportionate reductions in executive or emotional accessibility. Hysteresis describes asymmetric recovery, where returning to baseline functioning follows a different trajectory than entry into dysregulation. Recovery lag refers to the delayed restoration of adaptive access after autonomic, sensory, endocrine, or affective perturbation. Cumulative load reflects interactions between sleep loss, pain, uncertainty, sensory overload, chronic stress, and relational threat in reducing accessibility (McEwen, 1998; McEwen and Wingfield, 2003).

This formulation aligns the model with dynamic systems and biopsychosocial approaches while outlining a distinct focus: the state-dependent accessibility of adaptive capacities (Thelen and Smith, 1994). The model does not claim that physiology determines behavior in isolation. Instead, physiology is treated as an organizing variable that interacts continuously with learning history, context, cognition, culture, developmental history, and relational experience.

Unlike broader biopsychosocial and dynamic-systems frameworks, which describe multilevel interactions among biological, psychological, and environmental factors, ICM 2.0 prioritizes accessibility as the primary explanatory construct and central interpretive target. The model therefore emphasizes how physiological organization may shape the real-time availability of previously acquired capacities, rather than merely describing system interactions.

Physiological state occupies a central organizational role in this heuristic because the antecedents and consequences do not exert uniform effects across all conditions. An antecedent may facilitate adaptive functioning under one physiological state but restrict accessibility under another. Similarly, consequences may influence future functioning through learning processes as well as their effects on physiological organization, recovery, stress accumulation, perceived safety, and regulatory stability. Accordingly, the model proposes that physiological state functions as a dynamic organizing variable through which environmental events are interpreted, experienced, and integrated, thereby shaping the moment-to-moment accessibility of adaptive capacities.

## 5 The interoceptive communication model (ICM 2.0)

ICM 2.0 is a conceptual, state-dependent neurophysiological framework designed to organize the interactions among autonomic regulation, interoceptive processing, affective signaling, sensory integration, environmental context, biological load, and regulatory modulation and describe how they influence adaptive accessibility across physiological conditions (Barrett and Simmons, 2015; Craig, 2009; Critchley and Garfinkel, 2017; Khalsa et al., 2018; Nord and Garfinkel, 2022; Gomez Uncu and Morejón Rodríguez, 2026).

The model does not present a definitive neuroanatomical map. Rather, it serves as a heuristic for organizing the relationships among physiological processes that may influence accessibility to adaptive capacities. Accordingly, Route 1 and Route 2 are conceptual labels describing broad processing tendencies. They should not be interpreted as evidence that cognition, emotion, or behavior proceeds through two discrete, isolated, or mutually exclusive neural pathways. This distinction is emphasized to avoid mechanistic overinterpretation and acknowledge that the proposed routing architecture remains theoretical and requires empirical validation.

Route 1 refers to a survival-oriented organizational tendency in which physiological resources are preferentially allocated toward protection, threat management, and immediate adaptation, resulting in restricted accessibility. In contrast, Route 2 refers to a modulatory organizational tendency characterized by interoceptive integration, physiological stability, executive modulation, reflective evaluation, and flexible adaptation. These tendencies overlap, fluctuate, and co-occur within the same individual across different contexts and time points. Consequently, they are understood dimensionally rather than categorically. The primary purpose of these organizational tendencies is not to classify individuals into fixed physiological categories. Instead, they provide a conceptual framework for understanding how fluctuations in physiological organization may influence moment-to-moment accessibility to adaptive capacities across contexts. Figure 1 presents an overview of the ICM 2.0.

**Figure 1:**
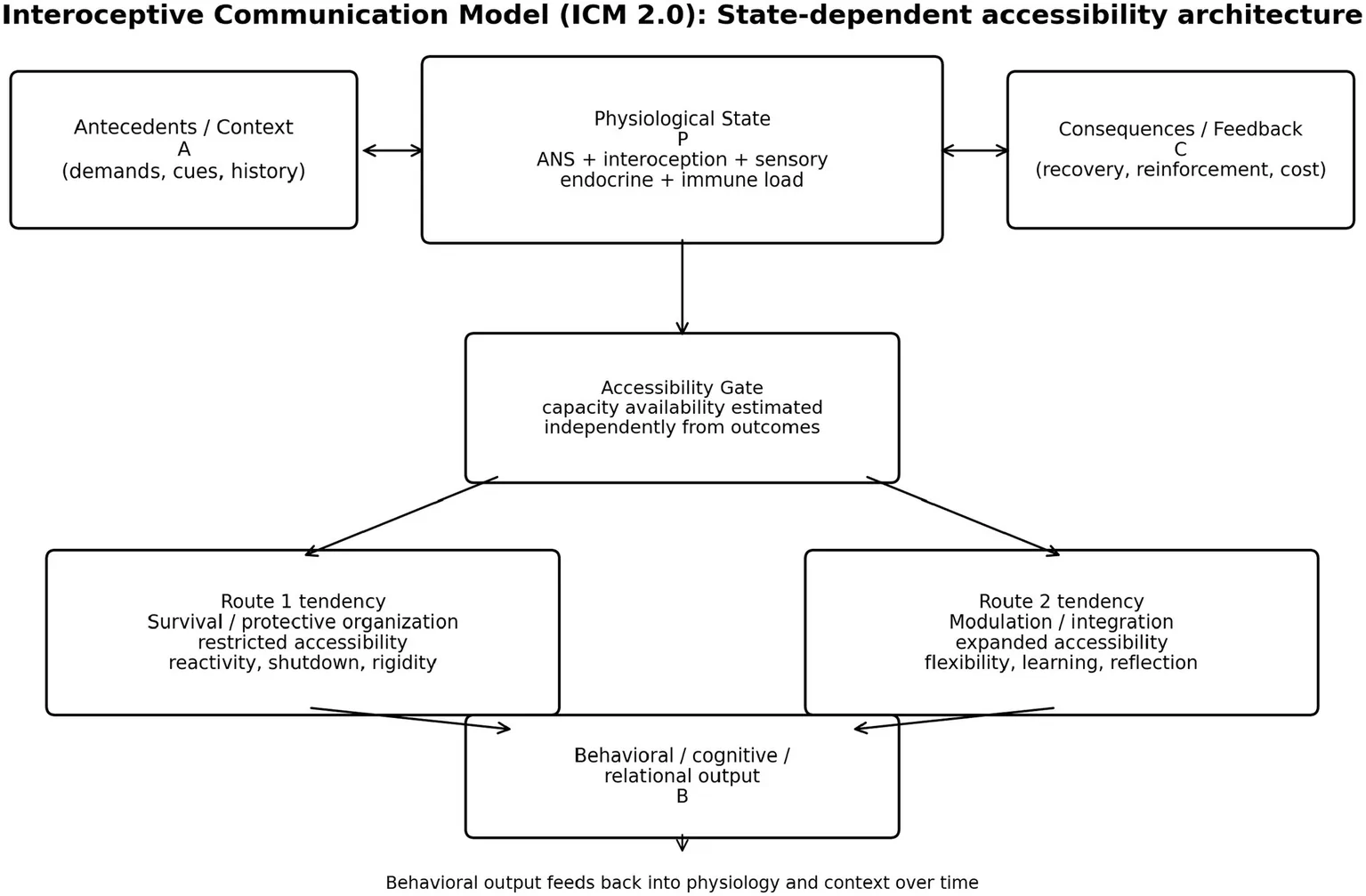
Conceptual overview of ICM 2.0 as a state-dependent accessibility architecture. A, antecedents; P, physiological state; C, consequences/feedback; B, behavioral, cognitive, emotional, or relational outputs. The figure is intended solely as a conceptual representation and should not be interpreted as a definitive neuroanatomical diagram.

## 6 Route 1: limbic-autonomic survival routing

Route 1 is conceptualized as a state-dependent tendency associated with automatic protective organization under conditions that are perceived, experienced, or physiologically registered as threatening, overwhelming, unpredictable, destabilizing, or insufficiently regulated. Under these conditions, physiological organization prioritizes immediate protection and survival, often resulting in reduced accessibility to reflective learning, behavioral flexibility, executive functioning, and sustained relational engagement (Porges, 2011; Lanius et al., 2018; Lanius et al., 2020).

Potential markers associated with Route 1 include heightened sympathetic activation, reduced vagally mediated flexibility, delayed recovery after stress, elevated skin conductance, respiratory dysregulation, sensory defensiveness, attentional narrowing, avoidance, freezing, shutdown, perseveration, impulsivity, or reduced error recovery (Agorastos et al., 2023; Kim et al., 2018; Mather and Thayer, 2018). However, these indicators should be interpreted probabilistically and contextually rather than as defensive markers of a particular routing tendency.

Route 1 is not be conceptualized as a discrete physiological state, diagnostic construct, or stable individual trait. Rather, it represents a theoretical tendency describing the conditions under which an individual’s physiological organization prioritizes protection over exploration, flexibility, learning, or reflective regulation. Individuals may transition fluidly between Routes 1 and 2 across situations, and characteristics associated with both routes may coexist simultaneously depending on contextual demands, physiological load, and available regulatory resources.

Furthermore, Route 1 is not inherently maladaptive. Rapid mobilization of protective physiological organization can be highly adaptive when responding to threats or destabilizing physiological demands. Difficulties may emerge when a survival-oriented organization becomes chronically activated, generalized to safe contexts, or be insufficiently modulated over time. Under such circumstances, adaptive functioning may remain constrained despite the individual’s possession of the relevant capacities.

## 7 Route 2: insular–prefrontal modulatory routing

Route 2 is conceptualized as a state-dependent processing tendency characterized by expanded interoceptive integration, autonomic flexibility, conscious evaluation, executive modulation, and accessibility to adaptive functioning. Within this organizational tendency, individuals are more likely to engage capacities associated with emotional regulation, attentional flexibility, planning, inhibitory control, adaptive learning, reflective processing, perspective taking, and sustained relational engagement (Craig, 2009; Critchley and Garfinkel, 2017; Khalsa et al., 2018; Thayer and Lane, 2000; Smith et al., 2017).

Potential indicators associated with Route 2 include greater autonomic flexibility, more efficient physiological recovery after perturbation, improved tolerance of sensory and emotional load, enhanced task initiation, reduced perseveration, increased metacognitive access, and improved use of previously acquired coping or learning strategies (Agorastos et al., 2023; Forte et al., 2019; Mather and Thayer, 2018). As with Route 1, these indicators are probabilistic rather than deterministic and should ideally be evaluated through multimethod assessment approaches rather than inferred solely from successful performance.

Importantly, Route 2 should not be viewed as a discrete physiological state, fixed endpoint of regulation, or stable individual characteristic. Instead, it represents a conceptual tendency describing conditions under which physiological organization supports expanded accessibility to adaptive behavioral, emotional, cognitive, relational, and regulatory capacities. Similar to Route 1, individuals may move fluidly between tendencies as physiological conditions and contextual demand change. Further, elements of both tendencies may coexist, reflecting the complex and multidimensional nature of physiological regulation.

Route 2 should not be idealized as a permanent or optimal state. Accessibility remains inherently dynamic and fluctuates in response to fatigue, illness, sleep disruption, relational context, environmental predictability, sensory demands, and accumulated physiological stress. Accordingly, ICM 2.0 emphasizes dynamic transitions and adaptation rather than fixed typologies.

## 8 State transitions and biological mechanisms

A central proposition of ICM 2.0 is that adaptive accessibility changes dynamically as physiological organization changes over time. Transitions between survival-oriented and modulatory tendencies may occur gradually or rapidly, depending on factors such as stress intensity, prior learning experiences, biological vulnerability, sensory load, relational context, and recovery capacity.

The biological mechanisms relevant to adaptive accessibility extend well beyond autonomic activation. These mechanisms include autonomic flexibility, respiratory regulation, neuroendocrine signaling, mediated through the hypothalamic–pituitary–adrenal axis, cortisol reactivity and recovery, sleep quality and fatigue, pain, inflammatory and neuroimmune signaling, metabolic state, sensory load, and the calibration of stress-response systems (Agorastos et al., 2023; Alotiby, 2024; Herman et al., 2012; James et al., 2023; Khalsa et al., 2018). Within the model, these systems are not proposed as separate causes of behavior. Rather, they are viewed as interacting physiological and contextual conditions that constrain or expand accessibility to adaptive capacities.

Developmental processes may influence adaptive accessibility by shaping the calibration of stress-response systems. Early life experiences, chronic adversity, relational environments, sensory exposure, and repeated physiological demands may contribute to long-term developmental patterns of autonomic flexibility, neuroendocrine responsiveness, recovery capacity, and accessibility across physiological states in the future (Feldman, 2017; McEwen and Wingfield, 2003).

Stress physiology provides a useful example of these processes. Acute stress may temporarily mobilize resources, facilitating adaptive action, whereas chronic or recurrent stress exposure can increase allostatic load, alter cortisol rhythms, influence immune signaling, and reduce recovery capacity. Neuroimmune processes may further influence mood, fatigue, cognitive performance, and behavioral readiness. Thus, the physiological state is conceptualized as a multisystem organization rather than a unidimensional continuum of arousal (Alotiby, 2024; Herman et al., 2012; James et al., 2023; McEwen, 1998; McEwen and Wingfield, 2003).

ICM 2.0 predicts that recovery dynamics may be influential as an activation process in determining accessibility. Individuals may exhibit comparable levels of stress reactivity but differ substantially in the speed, efficiency, and completeness with which adaptive accessibility is restored following physiological challenges or perturbation. Accordingly, accessibility may depend not only on peak physiological load but also on the effectiveness of recovery processes across autonomic, endocrine, immune, sensory, and affective systems. From this perspective, recovery represents an active component of accessibility rather than merely returning to baseline functioning (Agorastos et al., 2023; James et al., 2023; Kim et al., 2018).

## 9 Distinction from existing models

ICM 2.0 shares conceptual overlap with Polyvagal Theory, predictive processing, active inference, interoceptive theories of emotion, neurovisceral integration, and biopsychosocial frameworks. However, the model is not intended to replace these approaches. Rather, its primary aim is the organization of relationships between physiological state and moment-to-moment accessibility of adaptive capacities while positioning accessibility as the principal explanatory target (Porges, 2011; Barrett, 2017; Barrett and Simmons, 2015; Friston, 2010; Seth and Friston, 2016; Thayer and Lane, 2000; Smith et al., 2017; Thelen and Smith, 1994) (Table 2).

**Table 2:** Differentiation of ICM 2.0 from related frameworks.

Theory/framework	Main focus	Accessibility	Physiological state	Distinct prediction or emphasis
Polyvagal Theory	Autonomic hierarchy, social engagement, defensive states, vagal regulation.	Accessibility is implicitly rather than explicitly defined; adaptive functioning may vary by autonomic state, but accessibility is not the central construct.	Central, especially in the vagal organization and defensive autonomic states.	ICM 2.0 does not adopt a fixed hierarchical model of autonomic states. It predicts state-dependent variation in the accessibility of behavioral, cognitive, emotional, and relational capacities using multi-system markers.
Predictive processing/EPIC	Interoceptive prediction, prediction error, affective construction, and body–brain inference.	Accessibility to adaptive capacities is not conceptualized as a separate construct and is not the main outcome variable.	Crucial, especially in interoceptive prediction and allostasis.	ICM 2.0 translates brain–body prediction concepts into testable accessibility predictions: previously learned capacities should vary with independent physiological-state markers.
Active inference	Actions and perceptions as the regulation of uncertainty/free energy through generative models.	Accessibility is indirectly relevant; adaptive action is modeled computationally, but accessibility of adaptive capacities is not the central clinical/behavioral construct.	Importance varies across formulations depending on the active-inference model.	ICM 2.0 places less emphasis on computational modeling and greater emphasis on clinical, educational, and behavioral measurement of accessibility of adaptive capacities across real-world physiological states.
Neurovisceral integration	Relationships among HRV, prefrontal regulation, autonomic flexibility, cognition, and emotional functioning.	Closely related through regulatory flexibility, which is closely related to access. However, the construct is not framed as capacity availability.	Central, particularly through vagally mediated HRV and cortico-autonomic integration.	ICM 2.0 incorporates autonomic flexibility while broadening analysis to include sensory, interoceptive, endocrine, immune, contextual, and relational load.
Biopsychosocial/dynamic-systems models	Multilevel interactions among biological, psychological, and social processes over time.	Accessibility may be implied but is rarely operationalized as state-dependent access to adaptive capacities.	Included as one component within a broader framework.	ICM 2.0 identifies accessibility as the primary outcome and proposes specific within-person predictions across physiological states.
ICM 2.0	State-dependent accessibility of adaptive behavioral, emotional, cognitive, relational, and regulatory capacities.	Accessibility is the central explanatory construct and should be measured independently of performance outcomes.	Central and explicitly multisystem, encompassing autonomic, interoceptive, sensory, endocrine, immune, recovery, and context.	Individuals may possess a capacity yet show diminished real-time access when physiological load crosses a threshold, recovery is delayed, or regulatory resources become insufficient.

This table highlights conceptual distinctions among related frameworks and does not imply that the listed theories are mutually exclusive. Framework descriptions are based on Porges (2011), Barrett (2017), Barrett and Simmons (2015), Friston (2010), Seth and Friston (2016), Thayer and Lane (2000), Smith et al. (2017), Thelen and Smith (1994), and Smith and Thelen (2003).

The explanatory gap addressed by ICM 2.0 concerns situations in which individuals possess adaptive capacities but access to them is inconsistent across physiological states. Existing frameworks do not typically operationalize or position the moment-to-moment accessibility of previously acquired behavioral, emotional, cognitive, relational, and regulatory capacities as the primary construct (Porges, 2011; Barrett and Simmons, 2015; Seth and Friston, 2016; Thayer and Lane, 2000; Smith et al., 2017; Thelen and Smith, 1994). In contrast, ICM 2.0 proposes accessibility as the primary explanatory construct and the principal outcome. Variability in functioning may reflect fluctuations in accessibility rather than differences in skill acquisition, motivation, reinforcement history, prediction error, or autonomic regulation. In particular, the possession of a capacity and access to that capacity are not necessarily equivalent. An individual may demonstrate the capacity under one physiological state but exhibit substantially reduced access under another, despite no meaningful change in underlying knowledge, learning history, or skill acquisition. Consequently, ICM 2.0 generates a specific prediction: independently measured physiological conditions influence the real-time availability of previously demonstrated capacities, even when learning history and task knowledge remain stable. If supported empirically, this prediction implies that variability in functioning may reflect changes in accessibility rather than deficits in acquisition, motivation, or capability.

## 10 Falsifiability and testable predictions

A central aspect of scientific utility is the capacity of a conceptual framework to generate predictions that can be empirically supported, challenged, or refuted. Accordingly, ICM 2.0 is presented as a falsifiable framework because it generates specific within-person predictions concerning the relationship between independently measured physiological states and the accessibility of previously demonstrated capacities. The framework further specifies the conditions under which these predictions may be supported, challenged, or be rejected (Friston, 2010; Seth and Friston, 2016; Thelen and Smith, 1994).

Findings that would support the model include the following:

- Demonstrable within-person reductions in executive, emotional, learning-related, or behavioral accessibility during independently measured physiological dysregulation, despite preservation of underlying task knowledge.
- Disproportionate declines in performance after physiological load exceeds a critical threshold, rather than a linear relationship between task difficulty and behavioral outcomes.
- Recovery of adaptive accessibility following regulation-oriented interventions, environmental stabilization, co-regulation, sensory adjustment, sleep improvement, or autonomic recovery, occurring before additional skill training.
- Greater predictive accuracy of adaptive accessibility from multi-system state markers compared with trait measures, diagnostic category, or prior learning history alone.

Findings that would weaken or challenge the model include the following:

- Failure to detect an association between independent physiological-state markers and within-person fluctuations in accessibility to previously demonstrated capacities.
- Stable accessibility across high- and low-load physiological states despite adequate measurement of confounding variables.
- Behavioral variability that is fully explained by motivation, reinforcement history, task difficulty, or skill acquisition, with no additional predictive value from physiological state.
- Regulation-oriented interventions that fail to improve accessibility unless new skills are directly taught.

Several research designs can be used to evaluate these predictions, including repeated-measures stress/recovery paradigms, ecological momentary assessment combined with monitoring of physiological state using wearables, task performance under controlled sensory or cognitive load, pre-post regulation-first intervention trials, and cross-lagged longitudinal models examining physiological state, accessibility, and behavior over time. The strongest tests separate capacity acquisition from capacity accessibility by first establishing that a skill is demonstrably present under regulated conditions and subsequently evaluating its availability systematically under varied physiological states (Agorastos et al., 2023; James et al., 2023; Khalsa et al., 2018).

Future empirical studies should also compare accessibility-based models against alternative explanations based on motivation, reinforcement history, executive-function deficits, autonomic regulation, or predictive-processing accounts to determine whether accessibility contributes meaningful explanatory value beyond existing frameworks.

## 11 Interdisciplinary implications

ICM 2.0 has implications for trauma studies, autism research, education, rehabilitation, psychotherapy, psychiatry, and behavior analysis because each of these frequently encounters substantial state-dependent variability in functioning. The framework suggests that intervention sequencing should consider physiological accessibility before increasing instructional, cognitive, or behavioral demands.

Within trauma-related contexts, the model may help explain why explicit insight does not necessarily translate into adaptive action during periods of threat activation or dissociative states. In autism and sensory-related conditions, the framework may help explain fluctuations in adaptive functioning as a function of sensory load, environmental predictability, fatigue, and regulatory stability. In educational settings, the model supports the view that access to learning depends partly on physiological conditions that facilitate attention, emotional stability, and cognitive flexibility. In applied behavior analysis and behavioral interventions, ICM 2.0 encourages the integration of physiological regulation, antecedent modification, environmental predictability, and co-regulation, along with skill- and contingency-based approaches (Lanius et al., 2018; Lanius et al., 2020; Mazefsky et al., 2013; Mazefsky et al., 2012; Kern and Clemens, 2007; Lane et al., 2014; Cooper et al., 2020).

Notably, ICM 2.0 does not propose that physiological accessibility is the sole determinant of intervention outcomes. Rather, the framework proposes that accessibility may influence the timing and conditions under which previously acquired capacities become available for learning, performance, or adaptation. This perspective acknowledges the continued importance of instruction, reinforcement, cognitive processes, environmental supports, and developmental history.

The practical principle “regulation before instruction” should therefore be framed as a hypothesis-generating intervention sequence rather than a universal rule (Gomez Uncu, 2026b). ICM 2.0 predicts that, for some individuals, improvements in physiological accessibility may increase the probability that previously acquired capacities become accessible and available for adaptive performance. For others, meaningful improvement may still require direct instruction, reinforcement, environmental redesign, cognitive intervention, or other forms of support.

## 12 Limitations and future directions

ICM 2.0 is best understood as a conceptual and integrative framework rather than an empirically validated mechanistic theory. Its central constructs require further conceptual refinement, operational definition, psychometric development, experimental evaluation, and replication across populations, contexts, and developmental periods. Moreover, the proposed dual-route terminology remains hypothetical and should not be interpreted as evidence for two discrete neuroanatomical pathways.

A significant limitation of the framework is the absence of standardized, psychometrically validated measures. Future research should therefore focus on the development and validation measures of accessibility indices using multimethod assessment strategies that integrate physiological markers, task-based measures, ecological observation, self-report, or caregiver-report, and longitudinal within-person designs. Author-developed instruments or internally derived assessment frameworks should be regarded as preliminary tools only until independent evidence for reliability, validity, sensitivity to change, and incremental predictive utility has been established (Garfinkel et al., 2015; Mehling et al., 2018; Murphy et al., 2019).

Another limitation concerns the potential risk of physiological reductionism. ICM 2.0 does not propose that behavior is determined solely by physiological processes. Learning history, environmental influences, cognition, development, culture, disability, relational context, trauma history, and social conditions remain essential determinants of functioning. Rather, the framework proposes that physiological organization interacts dynamically with these variables to influence what becomes accessible in real time.

Future studies should determine whether accessibility explains meaningful variance beyond established constructs such as arousal, engagement, motivation, readiness, executive functioning, symptom severity, autonomic regulation, and interoceptive processing. Ultimately, ICM 2.0 will be scientifically useful only if accessibility demonstrates incremental predictive validity and if the framework can be empirically distinguished from competing theoretical explanations.

## 13 Conclusion

The ICM 2.0 proposes that behavioral, emotional, cognitive, relational, and regulatory capacities are not uniformly accessible across contexts. Instead, their moment-to-moment accessibility is influenced by dynamic interactions among physiological states, interoceptive processing, autonomic regulation, sensory load, biological stress systems, environmental context, learning history, and higher-order regulatory processes (Barrett and Simmons, 2015; Critchley and Garfinkel, 2017; Khalsa et al., 2018; Thayer and Lane, 2000).

The principal contribution of the revised framework is the introduction and elaboration of the construct of accessibility as an operational and potentially measurable construct. ICM conceptualizes accessibility as a latent, state-dependent construct that reflects the real-time availability of adaptive capacities that may be present but temporarily difficult to recruit. By specifying measurement strategies and distinguishing accessibility from related constructs, the framework seeks to provide a more precise account of why adaptive functioning varies across contexts. Furthermore, by clarifying potential non-linearity in physiological–behavioral relationships, differentiating ICM 2.0 from existing theoretical frameworks, and generating explicit falsifiable predictions, the revised model provides a clearer and articulated foundation for empirical research. Consequently, the framework shifts accessibility from a largely implicit assumption to an explicit target of measurement, explanation, and prediction.

ICM 2.0 should be regarded as a conceptual foundation for empirical inquiry and hypothesis generation rather than a complete explanatory theory or empirically validated model. Its value depends on whether future research empirically demonstrates that physiological states predict accessibility independently of skill acquisition, motivation, trait differences, and task demands, whether accessibility provides explanatory value beyond related constructs, and whether regulation-oriented approaches can expand access to adaptive functioning in measurable ways.

## Data Availability

The original contributions presented in the study are included in the article/supplementary material, further inquiries can be directed to the corresponding author/s.
